# Readmissions after Hospitalization for Heart Failure, Acute Myocardial Infarction, or Pneumonia among Young and Middle-Aged Adults: A Retrospective Observational Cohort Study

**DOI:** 10.1371/journal.pmed.1001737

**Published:** 2014-09-30

**Authors:** Isuru Ranasinghe, Yongfei Wang, Kumar Dharmarajan, Angela F. Hsieh, Susannah M. Bernheim, Harlan M. Krumholz

**Affiliations:** 1 Center for Outcomes Research and Evaluation, Yale-New Haven Hospital, New Haven, Connecticut, United States of America; 2 Division of Cardiology, Department of Internal Medicine, School of Medicine, Yale University, New Haven, Connecticut, United States of America; 3 The Robert Wood Johnson Clinical Scholars Program, Department of Internal Medicine, School of Medicine, Yale University, New Haven, Connecticut, United States of America; 4 Health Policy and Management, School of Public Health, Yale University, New Haven, Connecticut, United States of America; South African Medical Research Council, South Africa

## Abstract

**Background:**

Patients aged ≥65 years are vulnerable to readmissions due to a transient period of generalized risk after hospitalization. However, whether young and middle-aged adults share a similar risk pattern is uncertain. We compared the rate, timing, and readmission diagnoses following hospitalization for heart failure (HF), acute myocardial infarction (AMI), and pneumonia among patients aged 18–64 years with patients aged ≥65 years.

**Methods and Findings:**

We used an all-payer administrative dataset from California consisting of all hospitalizations for HF (n = 206,141), AMI (n = 107,256), and pneumonia (n = 199,620) from 2007–2009. The primary outcomes were unplanned 30-day readmission rate, timing of readmission, and readmission diagnoses. Our findings show that the readmission rate among patients aged 18–64 years exceeded the readmission rate in patients aged ≥65 years in the HF cohort (23.4% vs. 22.0%, p<0.001), but was lower in the AMI (11.2% vs. 17.5%, p<0.001) and pneumonia (14.4% vs. 17.3%, p<0.001) cohorts. When adjusted for sex, race, comorbidities, and payer status, the 30-day readmission risk in patients aged 18–64 years was similar to patients ≥65 years in the HF (HR 0.99; 95%CI 0.97–1.02) and pneumonia (HR 0.97; 95%CI 0.94–1.01) cohorts and was marginally lower in the AMI cohort (HR 0.92; 95%CI 0.87–0.96). For all cohorts, the timing of readmission was similar; readmission risks were highest between days 2 and 5 and declined thereafter across all age groups. Diagnoses other than the index admission diagnosis accounted for a substantial proportion of readmissions among age groups <65 years; a non-cardiac diagnosis represented 39–44% of readmissions in the HF cohort and 37–45% of readmissions in the AMI cohort, while a non-pulmonary diagnosis represented 61–64% of patients in the pneumonia cohort.

**Conclusion:**

When adjusted for differences in patient characteristics, young and middle-aged adults have 30-day readmission rates that are similar to elderly patients for HF, AMI, and pneumonia. A generalized risk after hospitalization is present regardless of age.

*Please see later in the article for the Editors' Summary*

## Abstract 2

Isuru Ranasinghe and colleagues compare readmissions after hospitalization for heart failure, acute myocardial infarction, or pneumonia in adults aged 18 to 64 years with readmissions in those aged 65 and older.

*Please see later in the article for the Editors' Summary*

## Editors' Summary

**Background:**

Many elderly people who are admitted to hospital, successfully treated, and discharged are readmitted soon after, often for an unrelated illness. In the US, for example, nearly a fifth of Medicare beneficiaries are readmitted to hospital within 30 days of discharge (Medicare is a national insurance program that primarily pays for health care services for Americans aged 65 and older). Experts have recently coined the term “post-hospital syndrome” for the transient period of increased susceptibility to a range of adverse health events that elderly patients seem to experience and have suggested that exposure to stress during hospital stays may underlie the syndrome. For example, hospital patients frequently have their sleep disrupted because of hospital routines, they are often in pain, they may have insufficient food intake (sometimes because they are waiting for an operation), and they may lose physical conditioning because they are confined to bed. These and other stressors can reduce individuals' natural reserves and increase their vulnerability to a range of illnesses and conditions.

**Why Was This Study Done?:**

Although stress is one possible determinant of the post-hospital syndrome, the underlying causes and patterns of hospital readmission are generally poorly understood. In particular, it is not known whether the post-hospital syndrome affects young and middle-aged patients as well as elderly patients. Importantly, a better understanding of the post-hospital syndrome is needed before effective strategies to reduce hospital readmissions can be developed. In this retrospective observational cohort study, the researchers compare readmission rates, timing, and diagnoses after hospitalization for heart failure (HF), acute myocardial infarction (AMI; heart attack), and pneumonia among patients aged 18–64 years living in California with readmission rates, timing, and diagnoses among patients aged 65 years or older hospitalized for the same conditions. A retrospective observational cohort study analyzes data that has been already been collected for a group (cohort) of people. Readmission is common among people of all ages who are admitted to hospital for HF, AMI, and pneumonia, and readmissions after hospitalization for these conditions among elderly Medicare patients are used in the US as a measure of hospital quality; hospitals with high readmission rates are subject to a Medicare reimbursement penalty.

**What Did the Researchers Do and Find?:**

The researchers used the Healthcare Cost and Utilization Project inpatient dataset for California to identify all the hospitalizations for HF, AMI, and pneumonia in California in 2007–2009 and to obtain data on the 30-day unplanned rehospitalization rate, timing of readmission, and readmission diagnoses for the identified patients (more than half a million patients). Nearly 30% of all hospital readmissions after hospitalization for HF, AMI, and pneumonia in California occurred among patients aged 18–64. After hospitalization for AMI, pneumonia, and HF, 11.2%, 14.4%, and 23.4%, respectively, of young and middle-aged patients were readmitted. Notably, the 30-day readmission rate among patients aged 18–64 admitted for HF exceeded the readmission rate among elderly patients admitted for the same condition. After allowing for other factors likely to affect the risk of readmission such as other illnesses, the 30-day readmission risk in patients aged 18–64 was similar to that in patients aged 65 years or older admitted for HF and pneumonia and only marginally lower among patients admitted for AMI. Finally, the timing of readmission was similar in both age groups and diagnoses other than the index admission diagnosis accounted for a substantial proportion of readmissions in both age groups.

**What Do These Findings Mean?:**

This study shows that after adjusting for differences in patient characteristics, the 30-day hospital readmission rates among young and middle-aged patients after hospitalization for HF, AMI, and pneumonia were similar to those among elderly patients. Moreover, the timing of readmission and the reasons for readmission among young and middle-aged patients were similar to those among elderly patients. These findings may not apply to other US states or to other countries and may not reflect the pattern of hospital readmissions following conditions other than HF, AMI, and pneumonia. Nevertheless, these findings suggest that the post-hospital syndrome affects young and middle-aged as well as elderly patients. Hospital readmission should therefore be considered as a potential problem for people of all ages and broad-based, multidisciplinary strategies that target patients of all ages should be developed to mitigate the risk of hospital readmissions.

**Additional Information:**

Please access these websites via the online version of this summary at http://dx.doi.org/10.1371/journal.pmed.1001737.

- The Institute for Healthcare Improvement provides information about reducing avoidable hospital readmissions
- Information about the US Centers for Medicare & Medicaid Services readmissions reduction program is available
- An article written by one of the study authors about the post-hospital syndrome is available

## Introduction

Reducing readmissions is a key focus of current health policy initiatives in the United States (US). The scope of the problem among elderly individuals is well established—one in five Medicare beneficiaries are readmitted within 30 days of discharge, with high cost to the health care system [1]. Hospitalizations, however, are not limited to elderly patients; many young and middle-aged adults aged 18–64 years are also hospitalized. For example, among the conditions that form the basis of current Medicare readmission measures [2]–[4], patients younger than 65 years of age account for approximately 30% of heart failure (HF) admissions [5],[6], 40–45% of acute myocardial infarction (AMI) admissions [7], and 33% of pneumonia admissions [8], and they consume 32% to 44% of aggregated costs for these conditions [9]. Furthermore, while elderly patients aged ≥65 years represent 12.9% of the population, young adults represent nearly 60% of the US population [10]. However, compared with elderly patients, our understanding of readmission risks in these younger patients is limited.

This information can help in ascertaining whether the phenomenon of vulnerability after hospital discharge is primarily limited to older patients. Elderly patients experience a period after hospitalization when they are more likely to be readmitted for an array of conditions often unrelated to the index admission diagnosis or the severity of index illness, with the risk highest immediately post-discharge [11]. This increased susceptibility has been referred to as post-hospital syndrome [12]. Whether such a period of increased susceptibility also occurs in young patients is uncertain. Older age is associated with comorbidities, frailty, cognitive and functional impairment, polypharmacy, and social isolation, all of which increase the risk of morbidity. If the post-hospital syndrome is limited to elderly patients, who have these distinctive age-related features, we would expect readmission rates to be lower among those of younger age. Furthermore, if an acquired period of generalized risk is absent in young patients, then we would expect a greater percentage of readmissions to be related to the initial admission diagnosis rather than due to acquired conditions. Although publicly available reports indicate a lower 30-day readmission rate of 5–11% across all conditions in young patients [13],[14], these brief reports have examined neither readmission rates across ages for the same conditions nor the diagnoses or timing of readmissions. Thus, it is difficult to conclude whether a post-hospital syndrome extends to patients younger than 65 years of age.

Distinguishing the timing and patterns of readmission seen in the young from those who are older may improve transitional care more broadly and facilitate more effective initiatives to reduce readmissions. Recent policy measures [15],[16] have incentivized interventions to reduce readmissions, yet variable conclusions have been drawn about the effectiveness of existing interventions [17]–[19]. This may reflect a lack of in-depth understanding of the underlying determinants of readmission. Many interventions also target elderly patients [18] and rarely address readmissions in younger patients. Understanding the conditions with which young patients present, and the time frame within the 30 days during which these occur, may inform whether specific interventions for younger patients are required.

Using an all-payer dataset from California, we compared the rate, timing, and readmission diagnoses following hospitalization for HF, AMI, and pneumonia between young and middle-aged adults aged 18–64 years and those aged ≥65 years. We chose HF, AMI, and pneumonia, because these conditions are frequent causes of readmissions among both young and elderly patients [13],[20]. HF, AMI, and pneumonia readmissions among Medicare patients aged ≥65 are publicly reported in the US as a measure of hospital quality [2]–[4] and have been extensively studied. Given that we understand the rate, timing and readmission diagnoses among elderly patients with HF, AMI, and pneumonia [11], patients with these conditions form an ideal population in which to perform a comparison with younger patients.

## Methods

### Ethics Statement

Institutional review board approval for this study, including waiver of the requirement for participant informed consent, was obtained from the Yale University Human Investigation Committee.

### Study Population

We identified hospitalizations from California included in the all-payer Healthcare Cost and Utilization Project (HCUP) state inpatient dataset and derived three separate cohorts of hospitalizations with a principal discharge diagnosis of HF, AMI, or pneumonia between January 2007 and November 2009. HF, AMI, and pneumonia were defined with International Classification of Diseases, Ninth Revision Clinical Modification (ICD-9-CM) codes identical to those used in the Centers for Medicare & Medicaid Services (CMS) publicly reported readmission measures [2]–[4] (see Table S1). Hospitalizations for patients aged ≥18 years from each cohort were included. In alignment with the CMS measures, we excluded hospitalizations during which a patient died, was transferred to another hospital, or was discharged against medical advice. We excluded hospitalizations for non-California residents since potential readmissions may not be captured in the state inpatient dataset. Hospitalization occurring after 30 days from previous discharge was counted as a new index admission if hospitalizations met the inclusion criteria. All included hospitalizations were stratified into hospitalizations among young patients aged 18–64 years and those occurring in elderly patients aged ≥65 years. We further subdivided the 18–64 years age group into young (aged 18–39 years) and middle-aged adults (age 40–54 years and 55–64 years) to assess readmission patterns with decreasing age. We presented patient data grouped by these age categories for clarity of presentation. However, to further characterize the association between age and the risk of readmission, and to ensure that the age categories we used were appropriate, we evaluated the association between age and the risk of readmission modeling age as a continuous variable.

### Classification of Comorbidities and Readmission Diagnoses

Comorbidities at the index hospitalization were categorized using the CMS condition categories (CCs) model [21], which groups presenting diagnoses into clinically coherent conditions. In contrast, readmission diagnoses were classified using a modified version of the CC model that we previously developed [11] because 85% of the 189 CC groups each accounted for less than 1% of all readmissions. Modified CCs were derived by consolidating common readmission diagnoses into a shorter list of 30 categories. Cardiopulmonary diagnoses were described with greater detail given their importance following index hospitalization for HF, AMI, or pneumonia. These 30 modified CCs were designed to be clinically relevant, internally consistent, and capture the most common readmission diagnoses after discharge from HF, AMI, and pneumonia hospitalizations (see Table S2) [11].

### Outcomes Assessment

We identified the first unplanned readmissions due to any cause occurring within 30 days of hospitalization. In keeping with CMS criteria for reporting hospital readmission performance [2]–[4], we excluded planned hospitalizations as identified by the CMS planned readmission algorithm [22]. To determine the timing of readmission among readmitted patients, we calculated the percentage of readmissions occurring on each day (0–30) following discharge.

### Readmission Diagnoses

We observed the percentage of readmissions occurring due to each of the 30 modified CCs for HF, AMI, and pneumonia during the 30 days postdischarge. To determine the proportion of readmission diagnoses that related to the index hospitalization diagnosis, we also calculated the proportion of 30-day readmissions occurring due to cardiovascular diagnoses after hospitalizations for HF, AMI, and pulmonary diagnoses following hospitalizations for pneumonia by aggregating the modified CCs (modified CCs comprising cardiovascular and pulmonary diagnoses are listed in Table S3 and Table S4).

### Statistical Analysis

Data are summarized as frequencies and percentages for categorical variables. Continuous variables are presented as mean ± standard deviation. The χ^2^ statistic, Student's t-test and F-test in ANOVA model were used to compare characteristics between age groups as appropriate. The Cochran-Armitage test was used to assess trends across age groups. Risk of readmission following discharge was determined using Kaplan-Meier estimates and age groups were compared using the log-rank test. Risk-adjusted hazard ratios (HRs) with 95% confidence intervals (CIs) for readmission among age groups were derived using a Cox proportional hazards model with adjustment for sex, race, payer status, and comorbidities at index hospitalization. To determine if disease severity influences the risk of readmission, we performed a sensitivity analysis by further adjusting for the length of the index hospital stay as a surrogate for disease severity. We censored patients if they died or did not have a readmission within 30 days of discharge. To evaluate the association between age as a continuous variable and the outcome of readmission within 30 days of discharge, we first derived the fracpoly logistic regression models with 30-day readmission as the dependent variable and age as the main covariate (independent variable), as well as with or without other confounding factors in the models. Then we calculated the unadjusted or adjusted readmission rates from the models with or without the confounding factors separately for each age value. Finally we plotted the association between the age and the unadjusted or adjusted readmission rates.

For all statistical analysis, significance levels were 2-sided with a P value<0.05. All analyses were conducted using SAS 9.2 (SAS Institute Inc, Cary, NC).

## Results

In California, for all conditions, 6,622,302 hospitalizations occurred among adult patients aged ≥18 years during the study period, which included 4,115,502 hospitalizations among patients aged 18–64 years and 2,506,800 hospitalizations among patients aged ≥65 years. Readmission within 30 days occurred following 10.3% (670,676) of hospitalizations with a readmission rate of 8.2% (336,513) among patients aged 18–65 years and 13.3% (334,163) among those aged ≥65 years. Among all hospitalizations, a total of 206,141 hospitalizations for HF, 107,256 hospitalizations for AMI, and 199,620 hospitalizations for pneumonia met our study inclusion criteria. Among these cohorts, 58,119 (28.2%) hospitalizations for HF, 42,546 (39.7%) hospitalizations for AMI, and 67,049 (33.6%) hospitalizations for pneumonia were among patients aged 18–64 years, while the remainder occurred in patients aged ≥65 years.

### Patient Characteristics at Index Hospitalization

The characteristics of the cohorts are described in Tables 1–3. The mean age of patients aged 18–64 years was 52.9 years for HF, 53.9 years for AMI, and 49.7 years for pneumonia, while the mean age of patients aged ≥65 years was approximately 80 years in each cohort. Patients aged 18–64 years were also more likely to be of nonwhite race and have private health insurance or Medicaid recorded as the payer. Among these patients, Medicare was recorded as a payer in 26.0% of HF hospitalizations, 12.3% of AMI hospitalizations, and 23.7% of pneumonia hospitalizations. In contrast, most patients aged ≥65 years were white and nearly 90% of patients in each cohort had Medicare health insurance. Comorbidities were generally less frequent among patients aged 18–64 years compared with elderly patients across all three cohorts, although this difference was less evident in the HF cohort. Across cohorts, comorbidities such as major psychiatric disorders and drug- and alcohol-related disorders were more common among patients aged 18–64 years and, as expected, comorbidities such as stroke (0.8–2.2%) and dementia (0.8–3.3%), were infrequently seen.

**Table 1:** Baseline characteristics of patients with an index hospitalization for heart failure.

Characteristic	Age Group (Years)				*p*-Value
	18–39	40–54	55–64	≥65	
**No. of hospitalizations**	4997	23500	29622	58119	
**Age, mean (SD)**	33.0 (5.0)	48.6 (4.0)	59.7 (2.8)	80.0 (7.7)	<0.001
**Male (%)**	65.7	64.1	58.6	45.2	<0.001
**Race (%)**					
White	26.7	34.8	42.2	62.0	<0.001
Black	26.3	30.8	22.0	8.4	
Hispanic	28.3	23.0	23.8	16.7	
Other	18.8	11.5	12.0	13.0	
**Payer status (%)**					
Medicare	15.6	21.9	31.1	88.6	<0.001
Medicaid	36.7	34.3	28.8	4.3	
Private insurance	22.8	23.7	28.8	6.3	
Other	24.9	20.2	11.3	0.7	
**Comorbidities at hospitalization (%)**					
Congestive heart failure	49.0	52.5	52.4	49.6	<0.001
Acute coronary syndrome	4.8	10.4	13.2	11.9	<0.001
Chronic atherosclerosis	19.4	42.0	57.6	65.0	<0.001
Stroke	1.4	2.0	2.5	2.6	<0.001
Diabetes and complications	28.5	47.9	61.8	47.7	<0.001
End-stage renal disease or dialysis	10.1	8.1	8.5	4.2	<0.001
Chronic obstructive pulmonary disease	13.9	31.8	38.9	38.0	<0.001
Pneumonia	24.1	24.6	26.4	29.4	<0.001
Asthma	17.9	12.9	9.0	5.8	<0.001
Dementia and senility	0.5	1.1	2.8	16.8	<0.001
Hemiplegia, paralysis, and functional disability	3.5	6.1	8.5	6.1	<0.001
Major psychiatric disorders	7.2	9.8	8.5	4.7	<0.001
Drug and alcohol abuse	47.8	50.8	36.0	11.1	<0.001

**Table 2:** Baseline characteristics of patients with an index hospitalization for acute myocardial infarction.

Characteristic	Age Group (Years)				*p*-Value
	18–39	40–54	55–64	≥65	
**No. of hospitalizations**	1979	17895	22672	64710	
**Age, mean (SD)**	34.4 (4.5)	48.8 (3.9)	59.6 (2.8)	78.3 (7.9)	<0.001
**Male (%)**	76.6	76.0	71.4	52.2	<0.001
**Race (%)**					
White	44.4	54.0	58.7	66.6	<0.001
Black	10.9	9.0	7.3	5.2	
Hispanic	25.5	20.6	19.1	14.5	
Other	19.2	16.4	14.9	13.8	
**Payer status (%)**					
Medicare	6.7	8.7	15.7	86.8	<0.001
Medicaid	17.3	15.0	13.2	3.2	
Private insurance	53.3	55.8	55.3	8.9	
Other	22.7	20.5	15.9	1.0	
**Comorbidities at hospitalization (%)**					
Congestive heart failure	5.8	6.0	8.9	16.6	<0.001
Acute coronary syndrome	5.5	6.8	8.2	10.8	<0.001
Chronic atherosclerosis	67.7	81.3	84.2	79.5	<0.001
Stroke	0.5	0.7	0.9	2.0	<0.001
Diabetes and complications	23.9	31.4	39.7	40.3	<0.001
End-stage renal disease or dialysis	2.7	2.0	2.9	2.1	<0.001
Chronic obstructive pulmonary disease	2.9	9.0	15.4	22.5	<0.001
Pneumonia	5.1	5.1	7.8	15.7	<0.001
Asthma	7.2	5.0	4.6	3.9	<0.001
Dementia and senility	0.2	0.4	1.2	14.9	<0.001
Hemiplegia, paralysis, and functional disability	1.6	2.1	3.0	4.1	<0.001
Major psychiatric disorders	3.0	3.8	3.7	3.4	0.020
Drug and alcohol abuse	4.3	4.2	3.7	2.1	<0.001

**Table 3:** Baseline characteristics of patients with an index hospitalization for pneumonia.

Characteristic	Age Group (Years)				*p*-Value
	18–39	40–54	55–64	≥65	
**No. of hospitalizations**	12288	26997	27764	132571	
**Age, mean (SD)**	30.3 (6.1)	48.2 (4.08)	59.6 (2.8)	80.0 (7.8)	<0.001
**Male (%)**	49.4	49.5	49.3	46.3	<0.001
**Race (%)**					
White	40.6	51.0	58.2	65.9	<0.001
Black	11.2	14.8	10.7	4.8	
Hispanic	30.2	21.8	19.4	14.9	
Other	18.0	12.5	11.7	14.4	
**Payer status (%)**					
Medicare	12.2	22.5	30.0	89.2	<0.001
Medicaid	31.3	26.7	23.2	4.3	
Private insurance	38.1	36.0	37.6	5.9	
Other	18.4	14.8	9.2	0.6	
**Comorbidities at hospitalization (%)**					
Congestive heart failure	5.3	10.9	16.1	21.4	<0.001
Acute coronary syndrome	0.5	2.3	3.8	4.4	<0.001
Chronic atherosclerosis	2.0	11.0	22.9	37.2	<0.001
Stroke	0.5	1.2	1.7	2.7	<0.001
Diabetes and complications	14.9	29.4	39.7	34.6	<0.001
End-stage renal disease or dialysis	2.8	3.5	4.3	2.2	<0.001
Chronic obstructive pulmonary disease	8.1	28.6	42.4	45.9	<0.001
Pneumonia	91.4	92.2	92.1	93.4	<0.001
Asthma	23.1	17.8	11.9	6.9	<0.001
Dementia and senility	1.9	2.4	4.8	26.0	<0.001
Hemiplegia, paralysis, and functional disability	8.6	6.5	7.3	6.4	<0.001
Major psychiatric disorders	8.1	13.3	12.8	6.8	<0.001
Drug and alcohol abuse	28.8	40.7	34.0	12.6	<0.001

### 30-Day Readmission Rates

Overall, there were 46,093 (22.4%) readmissions in the HF cohort, 16,117 (15.0%) readmissions in the AMI cohort, and 32,546 (16.3%) readmissions in the pneumonia cohort. The crude readmission rate in patients aged 18–64 years for the HF cohort exceeded the readmission rate observed among patients aged ≥65 years (23.4%, 95%CI 23.0%–23.7% versus 22.0%, 95%CI 21.8%–22.2%, *p*<0.001). In contrast, the crude readmission rate among patients aged 18–64 years was lower compared with patients aged ≥65 years for the AMI (11.2%, 95%CI 10.9%–11.5% versus 17.5%, 95%CI 17.2%–17.8%, *p*<0.001) and pneumonia (14.4%, 95%CI 14.1%–14.6% versus 17.3%, 95%CI 17.1%–17.5%, *p*<0.001) cohorts. Overall, approximately 30% of all 30-day readmissions occurred among patients 18–65 years of age (HF 29.5%, AMI 29.6%, pneumonia 29.6%).

When examining the trend across age groups (Table 4), the readmission rate in the HF cohort increased with progressively younger age groups. A readmission rate of 23.3% was observed in patients aged 18–39 years compared to a rate of 22.0% in patients aged ≥65 years (*p* for trend <0.001). In contrast, the 30-day readmission rate declined with progressively younger age groups in the AMI and pneumonia cohorts (both *p* for trend <0.001). A readmission rate of 9.3% (AMI) and 10.5% (pneumonia) was observed among patients aged 18–39 years in these cohorts.

**Table 4:** Hospitalization and readmissions among age groups for HF, AMI, and pneumonia.

Category	Age Group (Years)				Trend *p*-Value^a^
	18–39	40–54	55–64	≥65	
**Heart failure**					
Hospitalizations (n)	4997	23500	29622	148022	
Readmissions (n)	1165	5506	6911	32511	
Readmission rate (%, [95%CI])	23.3% [22.1–24.5]	23.4% [22.9–24.0]	23.3% [22.8–23.8]	22.0% [21.8–22.2]	<0.001
**Myocardial infarction**					
Hospitalizations (n)	1979	17895	22672	64710	
Readmissions (n)	184	1880	2712	11341	
Readmission rate (%, [95%CI])	9.3% [8.0–10.6]	10.5% [10.1–11.0]	12.0% [11.5–12.4]	17.5% [17.2–17.8]	<0.001
**Pneumonia**					
Hospitalizations (n)	12288	26997	27764	132571	
Readmissions (n)	1295	3854	4482	22915	
Readmission rate (%, [95% CI])	10.5% [10.0–11.1]	14.3% [13.9–14.7]	16.1% [15.7–16.6]	17.3% [17.1–17.5]	<0.001

a *p*-Value for trend (increase or decrease) across consecutive age groups (18–39, 40–54, 55–64, ≥65 years).

### Association of Age with Readmission Rates following Adjustment for Patient Characteristics

After adjustment for sex, race, payer status, and comorbidities, the adjusted risk of readmission in patients aged 18–64 years was similar to patients ≥65 years in the HF (HR 0.99; 95%CI 0.97–1.02) and pneumonia (HR 0.97; 95%CI 0.94–1.01) cohorts and marginally lower in the AMI cohort (HR 0.92; 95%CI 0.87–0.96).

**Figure 1:**
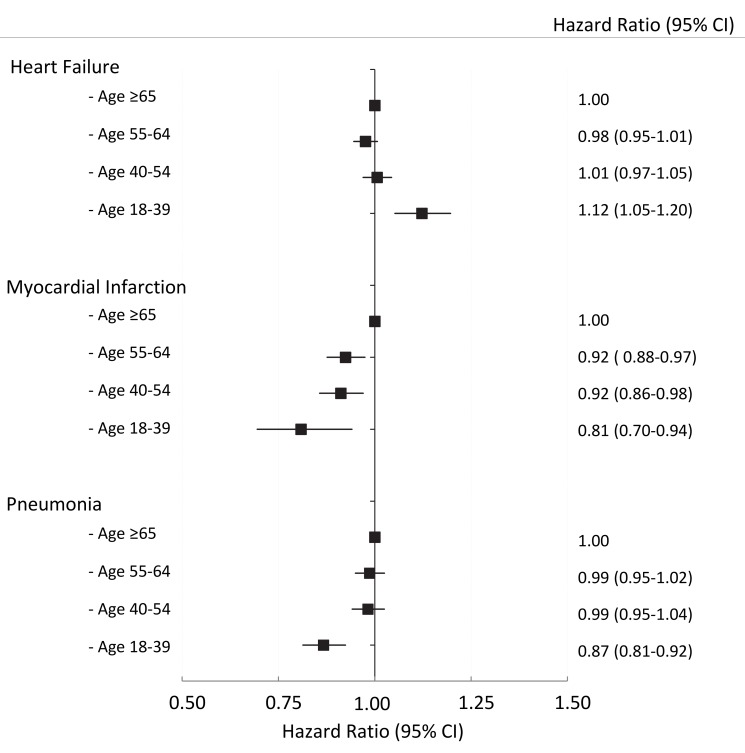
The adjusted risk of readmission by age group for HF, AMI, and pneumonia cohorts, respectively. The referent group is patients age ≥65 years within each cohort.

Figure 1 shows the adjusted risk of readmission among subgroups of younger patients compared with patients aged ≥65 years. In the HF cohort, the adjusted risk of readmission was similar among all subgroups of age except in those aged 18–39 years, who had a higher adjusted risk compared with patients aged ≥65 years (HR 1.12; 95%CI 1.05–1.20). In the AMI cohort, the adjusted risk of readmission declined with progressively younger age groups, with an adjusted HR of 0.81 (95%CI 0.70–0.94) in the youngest (18–39 year) age group. In the pneumonia cohort, the adjusted risk of readmission among subgroups of patients aged 18–64 years was similar to elderly patients except in the youngest (18–39 year) age group (HR 0.87; 95%CI 0.81–0.92). Further adjustment for the length of the index hospital stay (as a surrogate for disease severity) made no difference to the risk of readmission (see Table S5).

**Figure 2:**
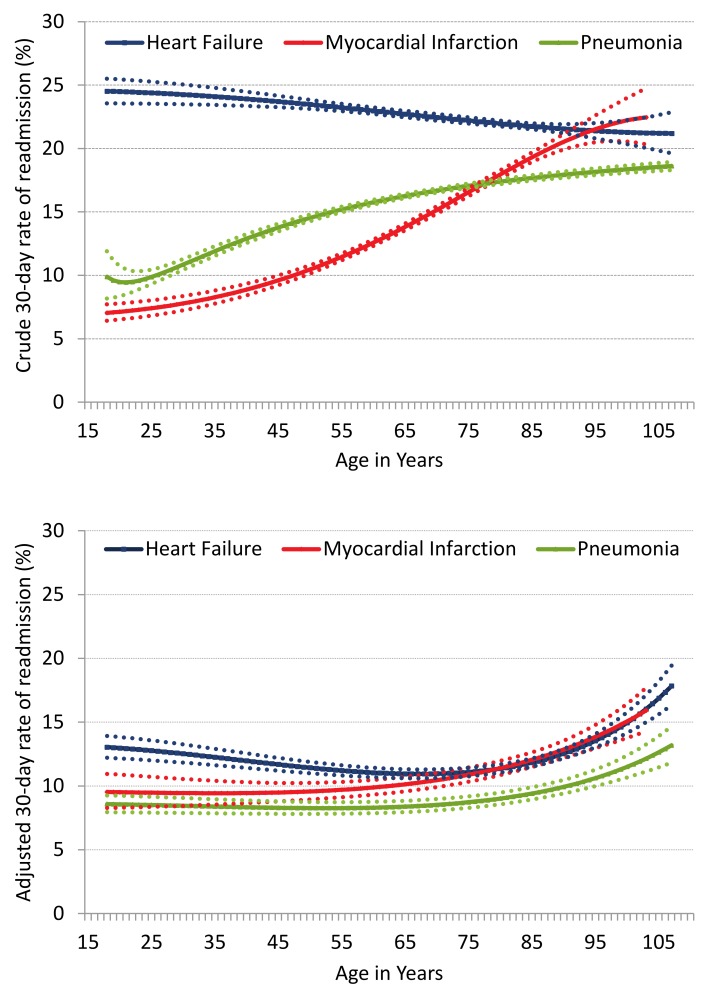
Crude and adjusted rate of readmission with increasing age for HF, AMI, and pneumonia. Age is modeled as a continuous variable using a flexible function. The dotted lines indicate the confidence intervals (5th and 95th percentile) for each cohort.

Figure 2 shows the association between age and the risk of readmission with age modeled as a continuous variable. The crude readmission rate varied considerably with age and disease condition. The adjusted rate of readmission for HF declined with increasing age, reaching a nadir at approximately 75 years of age before gradually increasing with increasing age thereafter. The adjusted rate of readmission for AMI shows a relatively stable rate among young patients but gradually rises after the age of 60–65 years. Similarly, for pneumonia, the adjusted rate of readmission was stable with increasing age until the age of 70–75 years after which the rate of readmission increases. Overall, the adjusted rates of readmission for the three cohorts were similar across a broad age range with the exception of upper extremes of age (>85 years) where the readmission rate rises rapidly with further acceleration above the age of 95 years.

### Timing and Risk of Readmission

**Figure 3:**
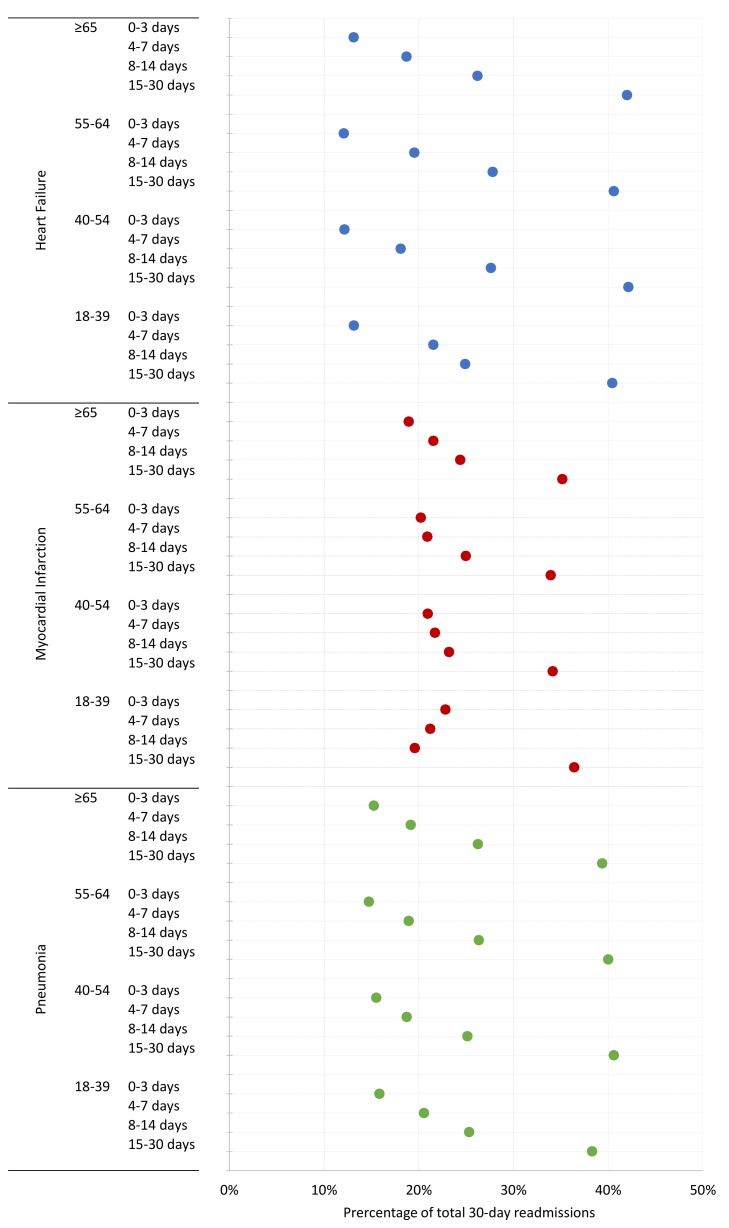
Percentage of readmissions by consecutive time periods following hospital discharge across age groups for HF, AMI, and pneumonia.

Figure 3 shows the proportion of patients readmitted within consecutive periods 0–3, 4–7, 8–14 and 15–30 days following discharge for each cohort. For all cohorts, most readmissions among patients aged 18–64 years occurred within 0–3 days (HF 13%, AMI 19%, pneumonia 15%) and 4–7 days (HF 19%, AMI 21%, pneumonia 19%). The proportion of young patients readmitted within each time period was similar to patients aged ≥65 years and was broadly similar across all subgroups of patients aged 18–64 years.

**Figure 4:**
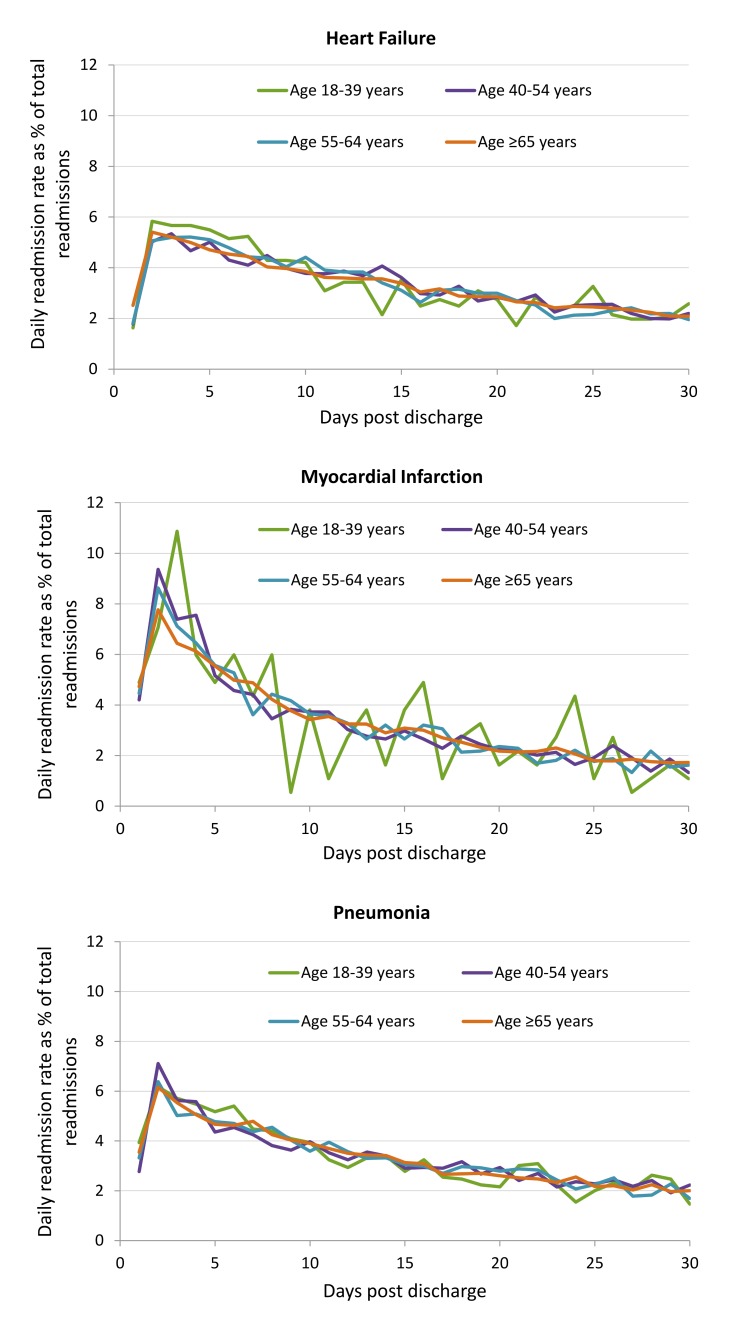
Readmission incidence rate by each day post-discharge by age group for HF, AMI, and pneumonia cohorts.

Figure 4 shows the percentage of 30-day readmissions occurring on each day postdischarge by age group for HF, AMI, and pneumonia cohorts. For all three cohorts, the risks of readmission were highest between days 2 and 5 after discharge. A gradual decline in the population risk was seen thereafter. There was no difference in risk of readmission by day postdischarge with age for any cohort as indicated by a significant overlap of curves for each age group.

### Readmission Diagnoses

The most frequent readmission diagnoses based on modified CCs by age group are shown in Table 5 (complete list by age group is given in Table 6 for HF, Table 7 for AMI, and Table 8 for pneumonia). In the HF cohort, HF was the most common principal readmission diagnosis in all age groups. With progressively younger age groups, HF was more frequently observed as the principal readmission diagnosis (increasing from 35.6% in those aged ≥65 years to 49.6% among those aged 18–39 years, *p* for trend <0.001). In the pneumonia cohort, pneumonia was the most common principal readmission diagnosis. With progressively younger age groups, pneumonia as a principal readmission diagnosis remained unchanged (22.2% in those aged ≥65 years to 23.2% in those aged 18–39 years, *p* for trend = 0.113). In the AMI cohort, HF was the principal readmission diagnosis among those aged ≥65 years. However, with progressively younger age groups, chest pain was observed to be the most frequent principal readmission diagnosis. Irrespective of age or cohort, a range of heterogeneous yet individually infrequent readmission diagnoses were present. For example, among 18–64-year-old patients in the HF cohort, 27 of the 30 modified CCs had a frequency <5% and a further 508 distinct primary ICD9-CM diagnoses (all with an individual frequency <1%) contributed to the “other” modified CC category.

**Table 5:** Principal diagnosis at readmission by age group for HF, AMI, and pneumonia.

Characteristic	Age Group (Years)								Trend *p*-Value^a^
	18–39		40–54		55–64		≥65		
	n	%	n	%	n	%	n	%	
**Heart failure**									
Heart failure	578	49.6	2489	45.2	2918	42.2	11569	35.6	<0.001
Renal disorders including renal failure	103	8.8	403	7.3	547	7.9	2676	8.2	0.131
Pneumonia including aspiration pneumonia	36	3.1	196	3.6	223	3.2	1750	5.4	<0.001
Septicemia/shock	17	1.5	120	2.2	209	3.0	1619	5.0	<0.001
Cardio-respiratory failure	32	2.7	183	3.3	255	3.7	1219	3.7	0.035
Readmission for a cardiac diagnosis^b^	716	61.5	3262	59.2	3892	56.3	16467	50.6	<0.001
**Myocardial infarction**									
Heart failure	13	7.1	215	11.4	386	14.2	2157	19.0	<0.001
Acute myocardial infarction	19	10.3	186	9.9	253	9.3	1124	9.9	0.790
Chest pain	38	20.7	321	17.1	269	9.9	431	3.8	<0.001
Complications of care	11	6.0	161	8.6	219	8.1	538	4.7	<0.001
Chronic angina and coronary artery disease	14	7.6	174	9.3	188	6.9	547	4.8	<0.001
Readmission for a cardiac diagnosis^b^	115	62.5	1175	62.5	1481	54.6	5992	52.2	<0.001
**Pneumonia**									
Pneumonia including aspiration pneumonia	301	23.2	812	21.1	849	18.9	5076	22.2	0.113
Septicemia/shock	73	5.6	236	6.1	384	8.6	2239	9.8	<0.001
Heart failure	59	4.6	237	6.1	277	6.2	1849	8.1	<0.001
Chronic obstructive pulmonary disease	59	4.6	327	8.5	437	9.8	1568	6.8	0.029
Cardio-respiratory failure	42	3.2	154	4.0	270	6.0	1189	5.2	<0.001
Readmission for a respiratory diagnosis^b^	461	35.6	1479	38.4	1753	39.1	8710	38.0	0.688

a *p*-Value for trend (increase or decrease) across consecutive age groups (18–39, 40–54, 55–64, ≥65 years).

b Readmission diagnosis grouped into cardiovascular and respiratory diagnoses based on criteria indicated in Tables S3 and S4.

**Table 6:** Principal readmission diagnosis within 30 days by modified condition categories among patients with an index hospitalization for heart failure.

Description	Age Group (Years)							
	18–39		40–54		55–64		≥65	
	n	%	n	%	n	%	n	%
Heart failure	578	49.6	2489	45.2	2918	42.2	11569	36.0
Acute myocardial infarction	5	0.4	80	1.5	134	1.9	783	2.4
Unstable angina and other acute ischemic heart disease	2	0.2	19	0.4	15	0.2	39	0.1
Chronic angina and coronary artery disease	3	0.3	52	0.9	105	1.5	323	1.0
Valvular/rheumatic heart disease	3	0.3	26	0.5	42	0.6	385	1.2
Other cardiac disease including congenital heart and hypertensive disease	24	2.1	60	1.1	62	0.9	216	0.7
Arrhythmias and conduction disorders	16	1.4	121	2.2	195	2.8	1001	3.1
Pleural effusion/pneumothorax	5	0.4	19	0.4	37	0.5	222	0.7
Chest pain	50	4.3	220	4.0	156	2.3	383	1.2
Syncope	4	0.3	12	0.2	30	0.4	210	0.7
Acute stroke/transient ischemic attack	11	0.9	80	1.5	101	1.5	768	2.4
Pulmonary embolism/deep venous thrombosis	15	1.3	38	0.7	41	0.6	159	0.5
Other peripheral vascular disease	5	0.4	65	1.2	93	1.4	631	1.9
Cardio-respiratory failure	32	2.8	183	3.3	255	3.7	1219	3.8
Chronic obstructive pulmonary disease	11	0.9	198	3.6	236	3.4	941	2.9
Pneumonia including aspiration pneumonia	36	3.1	196	3.6	223	3.2	1750	5.4
Septicemia/shock	17	1.5	120	2.2	209	3.0	1619	5.0
Urinary tract infection	3	0.3	18	0.3	44	0.6	587	1.8
Cellulitis	6	0.5	69	1.3	75	1.1	255	0.8
*Clostridium difficile*-associated infection	1	0.1	7	0.1	24	0.4	292	0.9
Renal disorders including renal failure	103	8.8	403	7.3	547	7.9	2676	8.2
Anemia	4	0.3	15	0.3	34	0.5	274	0.8
Gastrointestinal hemorrhage	4	0.3	37	0.7	81	1.2	632	1.9
Diabetes and its complications	27	2.3	119	2.2	169	2.5	559	1.7
Fibrosis of lung and other chronic lung disorders	2	0.2	10	0.2	14	0.2	91	0.3
Hip fracture	0	0.0	6	0.1	22	0.3	287	0.9
Complications of care	39	3.4	203	3.7	258	3.7	834	2.6
Other lung disorders including acute, congenital, and unspecified lung abnormalities	4	0.3	19	0.4	21	0.3	89	0.3
Primary cancer of the trachea, bronchus, lung, and pleura	0	0.0	3	0.1	3	0.0	58	0.2
Other^a^	155	13.3	619	11.2	767	11.1	3659	11.3

a Among patients aged 18–64 years, the “other” modified CC consisted of 508 distinct ICD-9-CM diagnoses. In comparison, among patients aged ≥65, the “other” modified CC consisted of 794 distinct ICD-9-CM diagnoses.

**Table 7:** Principal readmission diagnosis within 30 days by modified condition categories among patients with an index hospitalization for myocardial infarction.

Description	Age Group (Years)							
	18–39		40–54		55–64		≥65	
	n	%	n	%	n	%	n	%
Heart failure	13	7.1	215	11.4	386	14.2	2157	19.0
Acute myocardial infarction	19	10.3	186	9.9	253	9.3	1124	9.9
Unstable angina and other acute ischemic heart disease	14	7.6	84	4.5	76	2.8	234	2.1
Chronic angina and coronary artery disease	14	7.6	174	9.3	188	6.9	547	4.8
Valvular/rheumatic heart disease	0	0.0	2	0.1	6	0.2	68	0.6
Other cardiac disease including congenital heart and hypertensive disease	4	2.2	44	2.3	38	1.4	89	0.8
Arrhythmias and conduction disorders	5	2.7	39	2.1	76	2.8	423	3.7
Pleural effusion/pneumothorax	1	0.5	11	0.6	34	1.3	54	0.5
Chest pain	38	20.7	321	17.1	269	9.9	431	3.8
Syncope	1	0.5	18	1.0	26	1.0	114	1.0
Acute stroke/transient ischemic attack	3	1.6	34	1.8	64	2.4	336	3.0
Pulmonary embolism/deep venous thrombosis	2	1.1	30	1.6	29	1.1	146	1.3
Other peripheral vascular disease	2	1.1	28	1.5	70	2.6	253	2.2
Cardio-respiratory failure	3	1.6	24	1.3	59	2.2	359	3.2
Chronic obstructive pulmonary disease	2	1.1	25	1.3	42	1.6	217	1.9
Pneumonia including aspiration pneumonia	3	1.6	40	2.1	82	3.0	543	4.8
Septicemia/shock	4	2.2	40	2.1	86	3.2	580	5.1
Urinary tract infection	3	1.6	13	0.7	27	1.0	221	2.0
Cellulitis	2	1.1	10	0.5	21	0.8	53	0.5
*Clostridium difficile*-associated infection	0	0.0	3	0.2	11	0.4	127	1.1
Renal disorders including renal failure	4	2.2	53	2.8	117	4.3	606	5.3
Anemia	0	0.0	6	0.3	21	0.8	99	0.9
Gastrointestinal hemorrhage	1	0.5	21	1.1	53	2.0	333	2.9
Diabetes and its complications	4	2.2	34	1.8	46	1.7	167	1.5
Fibrosis of lung and other chronic lung disorders	0	0.0	3	0.2	5	0.2	10	0.1
Hip fracture	0	0.0	0	0.0	4	0.2	71	0.6
Complications of care	11	6.0	161	8.6	219	8.1	538	4.7
Other lung disorders including acute, congenital, and unspecified lung abnormalities	2	1.1	6	0.3	14	0.5	24	0.2
Primary cancer of the trachea, bronchus, lung, and pleura	0	0.0	3	0.2	8	0.3	22	0.2
Other^a^	29	15.8	252	13.4	382	14.1	1395	12.3

a Among patients aged 18–64 years, the “other” modified CC consisted of 298 distinct ICD-9-CM diagnoses. In comparison, among patients aged ≥65, the “other” modified CC consisted of 480 distinct ICD-9-CM diagnoses.

**Table 8:** Principal readmission diagnosis within 30-days by modified condition categories among patients with an index hospitalization for pneumonia.

Description	Age Group (Years)							
	18–39		40–54		55–64		≥65	
	n	%	n	%	n	%	n	%
Heart failure	59	4.6	237	6.2	277	6.2	1849	8.1
Acute myocardial infarction	1	0.1	24	0.6	54	1.2	299	1.3
Unstable angina and other acute ischemic heart disease	1	0.1	5	0.1	6	0.1	16	0.1
Chronic angina and coronary artery disease	1	0.1	9	0.2	25	0.6	68	0.3
Valvular/rheumatic heart disease	0	0.0	7	0.2	7	0.2	67	0.3
Other cardiac disease including congenital heart and hypertensive disease	12	0.9	30	0.8	24	0.5	116	0.5
Arrhythmias and conduction disorders	7	0.5	26	0.7	53	1.2	445	1.9
Pleural effusion/pneumothorax	26	2.0	73	1.9	65	1.5	272	1.2
Chest pain	13	1.0	83	2.2	67	1.5	208	0.9
Syncope	1	0.1	11	0.3	15	0.3	118	0.5
Acute stroke/transient ischemic attack	6	0.5	24	0.6	43	1.0	422	1.8
Pulmonary embolism/deep venous thrombosis	32	2.5	65	1.7	53	1.2	285	1.2
Other peripheral vascular disease	4	0.3	30	0.8	63	1.4	302	1.3
Cardio-respiratory failure	42	3.2	154	4.0	270	6.0	1189	5.2
Chronic obstructive pulmonary disease	59	4.6	327	8.5	437	9.8	1568	6.8
Pneumonia including aspiration pneumonia	301	23.2	812	21.1	849	18.9	5076	22.2
Septicemia/shock	73	5.6	236	6.1	384	8.6	2239	9.8
Urinary tract infection	18	1.4	37	1.0	50	1.1	508	2.2
Cellulitis	10	0.8	51	1.3	49	1.1	158	0.7
*Clostridium difficile*-associated infection	11	0.9	39	1.0	54	1.2	619	2.7
Renal disorders including renal failure	54	4.2	141	3.7	217	4.8	1189	5.2
Anemia	5	0.4	18	0.5	42	0.9	232	1.0
Gastrointestinal hemorrhage	16	1.2	31	0.8	59	1.3	434	1.9
Diabetes and its complications	28	2.2	48	1.3	70	1.6	278	1.2
Fibrosis of lung and other chronic lung disorders	16	1.2	48	1.3	48	1.1	251	1.1
Hip fracture	2	0.2	10	0.3	19	0.4	255	1.1
Complications of care	51	3.9	148	3.8	138	3.1	434	1.9
Other lung disorders including acute, congenital, and unspecified lung abnormalities	17	1.3	32	0.8	29	0.7	116	0.5
Primary cancer of the trachea, bronchus, lung, and pleura	0	0.0	33	0.9	55	1.2	238	1.0
Other^a^	429	33.1	1065	27.6	960	21.4	3664	16.0

a Among patients aged 18–64 years, the “other” modified CC consisted of 679 distinct ICD-9-CM diagnoses. In comparison, among patients aged ≥65, the “other” modified CC consisted of 838 distinct ICD-9-CM diagnoses.

When readmission principal diagnoses were more broadly grouped, a cardiovascular diagnosis accounted for a higher proportion of readmissions among patients aged 18–64 years compared with those aged ≥65 years in the HF and AMI cohorts. With progressively younger age groups, the proportion of cardiovascular readmissions increased for both HF and AMI cohorts (both *p* for trend <0.001), suggesting less diversity in readmission diagnoses in younger patients. In contrast, in the pneumonia cohort, a respiratory diagnosis accounted for 38.3% of all readmissions among patients aged 18–64 years. This finding is similar to those aged ≥65 (38.0%) and unchanged with progressively younger age groups (*p* for trend = 0.7).

Nevertheless, among younger patient age groups, a noncardiac diagnosis consistently represented 40–44% of readmissions in the HF cohort and 35–45% of readmissions in the AMI cohort. Similarly, a nonpulmonary diagnosis was present in 61–64% of patients in the pneumonia cohort. This suggests a diverse array of readmissions unrelated to the index hospitalization among young patients aged 18–64 years.

## Discussion

We observed that patients aged 18–64 years had a high rate of 30-day readmission following hospitalization. In these patients, 11.2% were readmitted after an index hospitalization for AMI, 14.4% were readmitted after an index hospitalization for pneumonia, and 23.4% were readmitted after an index hospitalization for HF. Indeed, in the HF cohort, the 30-day readmission rate in patients aged 18–64 years consistently exceeded the readmission rate seen in elderly patients. Following adjustment for patient characteristics other than age, young and middle-aged adults had 30-day readmission rates that were similar to elderly patients. The timing of readmission in patients aged 18–64 years was similar to patients aged ≥65 years, and, like elderly patients [11], the risk of readmission among these patients was highest immediately after discharge and progressively declined thereafter. While the readmission diagnoses were less diverse, many of the conditions that younger patients presented with were also unrelated to the index hospitalization akin to the well-documented readmission patterns seen in elderly patients [1],[11],[23]–[25]. For HF, AMI, and pneumonia, our observations suggest that patients aged 18–64 years, like those aged ≥65 years, may also experience a post-hospital syndrome [11],[12], albeit to a lesser degree than seen in elderly patients [11],[12]. Furthermore, these observations suggest that the increased risk of readmission for a wide range of conditions is not an issue solely limited to elderly patients. Rather, our findings suggest that the risk of readmission for a diversity of causes is a pervasive issue experienced by adult patients of all ages following hospitalization.

Two studies have reported data relevant to readmission among young patients and our findings should be evaluated in the context of these studies. Sommers et al [14], using data from 5,805 patients, evaluated physician visits following hospital discharge, and reported an all-cause readmission rate of 5% among patients aged 21–44 years and 9.5% among those aged 45–64 years. A HCUP statistical brief also reported a 30-day readmission rate for nonobstetrical causes following any hospitalization of up to 10.7% in patients aged 21–64 years [13]. In this study, 25% of all readmissions among young patients occurred following an index hospitalization for circulatory or respiratory disease, with a 30-day readmission rate among these conditions of 11% and 10%, respectively [13]. Our study extends the literature by undertaking an in-depth consideration of readmission timing and diagnosis among younger patients for HR, AMI, and pneumonia, conditions that are among the most common circulatory and pulmonary conditions. To our knowledge this has not been previously examined in patients aged younger than 65 years.

In elderly patients, a transient period of acquired vulnerability, termed post-hospital syndrome, is present following hospitalization [11],[12]. Among elderly patients aged ≥65 years, the readmission rates for HF, AMI, and pneumonia are consistently high (18.3%–24.8%) [11]. In contrast, among younger patients aged 18–64 years, the observed readmission rates were more variable across cohorts and age groups. This variation between cohorts was more prominent in the youngest age group (18–39 years), where the readmission rate varied from 9.3% (AMI) to 23.3% (HF). The readmission diagnosis in patients 18–64 years of age was more likely to be related to the index admission diagnosis compared with elderly patients. While this is less consistent with an acquired vulnerability post-hospitalization, a noncardiac diagnosis nevertheless represented a large portion of readmissions in the HF (42.1%) and AMI (42%) cohorts, with a nonpulmonary diagnosis present in 61–64% of patients in the pneumonia cohort in patients aged 18–64 years. The underlying etiological and presentation characteristics for HF, AMI, and pneumonia do differ across age groups. For example, cardiomyopathies, myocarditis, and congenital abnormalities are associated with HF in young patients, whereas coronary disease and hypertension commonly cause HF in elderly patients [26]. These differences may explain the variation in readmission patterns across cohorts and within age groups. Such etiological differences in the cause of HF may explain why patients with HF aged 18–39 years had a higher rate of readmission compared with older patients with HF even when adjusted for other covariates. In contrast to these differences, striking similarities in timing and risk of readmission post-discharge were observed between young and elderly patients; irrespective of the index diagnosis or age, a quantitatively similar heightened risk of readmission appears to exist for these populations immediately after hospital discharge and gradually declines. Furthermore, the proportion of patients that re-present within each consecutive time period post-discharge was remarkably consistent across age groups. Both these findings are highly suggestive of an acquired risk post-hospitalization.

Our study raises questions regarding the possible contributing factors to such an acquired risk. Elderly patients are well known to experience age-related factors such as cognitive and functional impairment [27], social isolation [28], and polypharmacy [29],[30]. Hospitalization in this context has been associated with undesirable consequences including functional decline [31], heightened risk of delirium [32], and greater adverse effects from therapeutic agents [33]. In contrast, physiological and psychological effects of hospitalization on young adults are largely unknown. More importantly, the adjusted risk of readmission among patients aged 18–39 years was only marginally lower when compared with the elderly patients and indeed higher in the HF cohort. The high risk of readmission even among the very young (18–34 year) age group, who are least likely to experience age-related factors, suggests the presence of alternate determinants of readmission among these age groups.

Comorbidities and sociodemographic characteristics may contribute to the high risk of readmission among young patients. Comorbidities were common among young patients. While many young adults in the community are healthy, young patients who are ultimately hospitalized with HR, AMI, and pneumonia may carry a sizable comorbidity burden. Specifically, comorbidities such as drug and alcohol abuse and psychiatric conditions were more common among younger age groups and particularly among those with HF. Substance abuse and psychiatric disorders increase the risk of readmission and other adverse outcomes such as mortality following hospitalization for HF, AMI, and pneumonia [33]–[36]; these disorders may contribute to the high rate of readmission among younger patients. However, it is important to note that drug and alcohol abuse and psychiatric illness were not a common principal readmission diagnosis. Therefore, while these factors may act as contributing factors, these conditions per se do not appear to be responsible for readmissions. A sizable proportion of young patients in our cohort had Medicare as the payer for hospitalization charges. Patients aged <65 years are eligible for Medicare in the event of a disability or a chronic condition. These patients may have a high burden of disease compared with the general population and may explain the higher rate of readmission observed. Furthermore, hospitalized younger patients were more likely to be African American, Hispanic, or other non-white races; these patients have higher rates of readmission compared with white patients [36]. We observed that adjustment for the difference in comorbidities, payer status, and race resulted in a substantial change in the crude and adjusted rates observed in the HF cohort among younger patients (Figure 2), which may further suggest that these factors contribute importantly to the risk of readmission among younger patients. Moreover, lower socioeconomic status and poor access to health care are also known to contribute to readmissions [36]. We did not measure these factors although they may have contributed to the high rate of readmission among younger patients, in addition to the potential etiological differences. Further studies are required to evaluate the contribution of comorbidities and sociodemographic characteristics to readmission among younger patients, as they may provide targets for intervention to reduce readmissions among these patients.

Our findings have implications for initiatives aimed at reducing hospital readmissions and health care costs. First, recent US policy measures [15],[16] to reduce readmissions, including public reporting of HF, AMI, and pneumonia, primarily target elderly Medicare patients. Given that young patients form a sizable volume of patients with high health care expenditure [9], and contribute to 30% of all readmissions, extending these initiatives to encompass young patients may lead to improved quality of care and reduced health care expenditure. Second, recent policy changes such as the Hospital Readmissions Reduction Program have incentivized interventions to reduce readmissions by enforcing financial penalties on hospitals with high readmission rates [15]. Many of these targeted interventions are aimed at elderly patients [18],[19]. Our finding of a generalized risk of readmission, and broad yet predictable readmission diagnoses and timing, strongly suggests the need for development of more broad-based multidisciplinary strategies to mitigate this risk. Finally, further research should focus on better understanding the drivers of readmission in young patients. Understanding the psychological and physiological effects of hospitalization, and the contributing effects of comorbidities such as substance abuse, may guide the development of more effective interventions relevant to young patients.

Our study has important issues to consider. First, our data are derived from a single state (California). However, this is the most populous US state, with 37.2 million residents or 12.1% of the overall US population [10]. Furthermore, the overall rates of readmission seen among the elderly population in our study are consistent with rates of readmission reported in prior national studies [1],[11], indicating that the California population is likely to be representative of a national sample. Second, we analyzed patients with an index diagnosis of HF, AMI, and pneumonia, the measures that are publicly reported by CMS. These conditions may not reflect the patterns for other diagnoses. Third, out-of-hospital deaths may have occurred during the 30 days following the index hospitalization. Out-of-hospital deaths within 30 days may be disproportionately higher among older patients and may result in a lower observed rate of readmission among these patients. HCUP data do not capture out-of-hospital deaths post-index hospitalization, and we cannot assess the impact of the competing risk of mortality. However, the absolute number of readmissions exceeds the number of deaths occurring within the 30 days and we anticipate the effects of any potential bias to be small [2]–[4],[34],[35]. The declining risk of readmission observed with time following hospitalization reflects the decline in the risk for the population as a whole. However, the risk of readmission for an individual patient may vary based on specific patient characteristics and circumstances. Lastly, our analysis was based on administrative data rather than clinical data. However, HCUP data are widely used and have been shown to be accurate when validated against chart review data [36],[37].

### Conclusion

In our study, nearly 30% of all readmissions for heart failure, acute myocardial infarction, and pneumonia occurred in young and middle-aged adults. When adjusted for differences in patient characteristics, young and middle-aged adults had 30-day readmission rates that were similar to elderly patients. While readmission is often perceived as a problem among elderly patients, our data suggest that readmission should be considered as a broader issue that extends to all hospitalized patients. The post-hospital syndrome, a period of generalized risk after hospitalization, appears to be present regardless of age.

## Supporting Information

Table S1 International Classification of Diseases, Ninth Revision, Clinical Modification Codes Used to Define Heart Failure, Acute Myocardial Infarction, and Pneumonia Cohorts.
(DOCX)

Table S2 Modified Condition Category Constituents.
(DOCX)

Table S3 Modified Condition Category codes for cardiovascular diagnoses.
(DOCX)

Table S4 Modified Condition Category codes for pulmonary diagnoses.
(DOCX)

Table S5 Adjusted 30-day risk of readmission with the addition of length of index hospital stay as a measure of disease severity.
(DOCX)

## Abbreviations

AMI: acute myocardial infarction
CC: condition category
CI: confidence interval
CMS: Centers for Medicare & Medicaid Services
HCUP: Healthcare Cost and Utilization Project
HF: heart failure
HR: hazard ratio
ICD-9-CM: International Classification of Diseases, Ninth Revision Clinical Modification
