# Supplementary material for: Readmissions after Hospitalization for Heart Failure, Acute Myocardial Infarction, or Pneumonia among Young and Middle-Aged Adults: A Retrospective Observational Cohort Study
Source: PLoS Med. 2014 Sep 30;11(9):e1001737. doi: 10.1371/journal.pmed.1001737 (PMC4181962; doi:10.1371/journal.pmed.1001737)
Supplement: Table S1 — International Classification of Diseases, Ninth Revision, Clinical Modification Codes Used to Define Heart Failure, Acute Myocardial Infarction, and Pneumonia Cohorts. (DOCX) [file pmed.1001737.s001.docx]

| Table S1: International Classification Of Disease, Ninth Revision, Clinical Modification Codes Used To Define Heart Failure, Acute Myocardial Infarction, And Pneumonia Cohorts | |
| --- | --- |
|  |  |
| Cohort name | **Constituent ICD-9-CM codes** |
| Heart failure | 402.01, 402.11, 402.91, 404.01, 404.03, 404.11, 404.13, 404.91, 404.93, 428.xx |
| Acute myocardial infarction | 410.xx excluding those with 410.x2 (AMI, subsequent episode of care) |
| Pneumonia | 480.x, 481, 482.xx, 483.x, 485, 486, 487.0, and 488.11 |
| ICD-9-CM: International Classification of Diseases, Ninth Revision, Clinical Modification | |
