# Supplementary material for: Readmissions after Hospitalization for Heart Failure, Acute Myocardial Infarction, or Pneumonia among Young and Middle-Aged Adults: A Retrospective Observational Cohort Study
Source: PLoS Med. 2014 Sep 30;11(9):e1001737. doi: 10.1371/journal.pmed.1001737 (PMC4181962; doi:10.1371/journal.pmed.1001737)
Supplement: Table S2 — Modified Condition Category Constituents. (DOCX) [file pmed.1001737.s002.docx]

| Table S2: Modified Condition Category Constituents. | | | | |
| --- | --- | --- | --- | --- |
|  |  | |  | |
| Modified condition category number and name | **Constituent CMS**  **condition category codes** | | **Additional ICD-9-CM diagnostic codes** | |
| 1 - Heart failure | 80 | |  | |
| 2 - Acute myocardial infarction | 81 | |  | |
| 3 - Unstable angina and other acute ischemic heart disease | 82 | |  | |
| 4 - Chronic angina and coronary artery disease | 83, 84 | |  | |
| 5 - Valvular/rheumatic heart disease | 86 | |  | |
| 6 - Other cardiac disease including congenital heart and  hypertensive disease | 85, 87, 88, 94, 89, 90, 91 | |  | |
| 7 - Arrhythmias and conduction disorders | 92, 93 | |  | |
| 8 - Pleural effusion/pneumothorax | 114 | |  | |
| 9 - Chest pain | N/a (constituent ICD-9-CM codes on right) | | 786.50, 786.59 | |
| 10 - Syncope | N/a (constituent ICD-9-CM codes on right) | | 780.2 | |
| 11 - Acute stroke/transient ischemic attack | 95, 96, 97 | |  | |
| 12 - Pulmonary embolism/deep venous thrombosis | N/a (constituent ICD-9-CM codes on right) | | 415.1, 415.11, 415.19, 453.4, 453.40, 453.41, 453.42, 453.8 453.81, 453.82, 453.83, 453.84, 453.85, 453.86, 453.87, 453.89, 453.9 | |
| 13 - Other peripheral vascular disease | 104, 105, 106 (excluded ICD-9-CM codes on right) | | Exclude 415.1, 415.11, 415.19, 453.4, 453.40, 453.41, 453.42, 453.8 453.81, 453.82, 453.83, 453.84, 453.85, 453.86, 453.87, 453.89, 453.9 | |
| 14 - Cardio-respiratory failure | 77, 78, 79 | |  | |
| 15 - Chronic obstructive pulmonary disease/asthma | 108, 110 | |  | |
| 16 - Pneumonia including aspiration pneumonitis | 111, 112, 113 | |  | |
| 17 - Septicemia/shock | 2 | |  | |
| 18 - Urinary tract infection and urinary system complaints | 135 | |  | |
| 19 - Cellulitis | 152 | |  | |
| 20 - Clostridium difficile-associated infection | N/a (constituent ICD-9-CM codes on right) | | 8.45 | |
| 21 - Renal disorders including renal failure and fluid,  electrolyte, and acid-base abnormalities | 131, 23 | |  | |
| 22 - Anemia | 47 | |  | |
| 23 - Gastrointestinal hemorrhage | N/a (constituent ICD-9-CM codes on right) | | 531.0, 531.00, 531.01, 531.2, 531.20, 531.21, 531.40, 531.41, 531.6, 531.60, 531.61, 532.0, 532.00, 532.01, 532.2, 532.20, 532.21, 532.4, 532.40, 532.41, 532.6, 532.60, 532.61, 533.0, 533.00, 533.01, 533.2, 533.20, 533.21, 533.4, 533.40, 533.41, 533.6, 533.60, 533.61, 534.0, 534.00, 534.01, 534.2, 534.20, 534.21, 534.4, 534.40, 534.41, 534.6, 534.60, 534.61, 535.01, 535.11, 535.41, 535.51, 535.61, 562.02, 562.12, 569.3, 578, 578.0, 578.1, 578.9 | |
| 24 - Diabetes and its complications | 15, 16, 17, 18, 19, 20 | |  | |
| 25 - Fibrosis of lung and other chronic lung disorders | 109 | |  | |
| 26 - Hip fracture | 158 | |  | |
| 27 - Complications of care | 163, 164, 165 | |  | |
| 28 - Other lung disorders including acute, congenital,  and unspecified lung abnormalities | 115 (additional ICD-9-CM codes on right) | | 786.0, 786.00, 786.01, 786.02, 786.05, 786.06, 786.9 | |
| 29 - Primary cancer of the trachea, bronchus, lung,  and pleura | N/a (constituent ICD-9-CM codes on right) | | 162.9, 162.3, 162.5, 162.8, 162.2, 162.4 | |
| 30 - Other admitting diagnoses | See below | | See below | |
| Constituents of modified condition category 30 -  other admitting diagnoses |  |  | |  |
| Other infections | 1, 3, 4, 5, 6 |  | |  |
| Cancer | 7, 8, 9, 10, 11, 12, 13, 14 |  | |  |
| Endocrine/metabolic | 21, 22, 24 |  | |  |
| Liver disease | 25, 26, 27, 28, 29 |  | |  |
| Digestive (Gastrointestinal) | 30, 31, 32, 33, 34, 35, 36 (Excluded ICD-9-CM codes on right) | Exclude 531.0, 531.00, 531.01, 531.2, 531.20, 531.21, 531.40, 531.41, 531.6, 531.60, 531.61, 532.0, 532.00, 532.01, 532.2, 532.20, 532.21, 532.4, 532.40, 532.41, 532.6, 532.60, 532.61, 533.0, 533.00, 533.01, 533.2, 533.20, 533.21, 533.4, 533.40, 533.41, 533.6, 533.60, 533.61, 534.0, 534.00, 534.01, 534.2, 534.20, 534.21, 534.4, 534.40, 534.41, 534.6, 534.60, 534.61, 535.01, 535.11, 535.41, 535.51, 535.61, 562.02, 562.12, 569.3, 578, 578.0, 578.1, 578.9 | |  |
| Musculoskeletal | 37, 38, 39, 40, 41, 42, 43 |  | |  |
| Other hematologic | 44, 45, 46 |  | |  |
| Cognitive | 48, 49, 50 |  | |  |
| Substance abuse | 51, 52, 53 |  | |  |
| Psychiatric | 54, 55, 56, 57, 58, 59, 60 |  | |  |
| Development disorders | 61, 62, 63, 64, 65, 66 |  | |  |
| Spinal cord | 67, 68, 69 |  | |  |
| Neuromuscular | 70, 71, 72, 73, 74, 75, 76 |  | |  |
| Non-acute cerebrovascular disease | 98, 99, 100, 101, 102, 103 |  | |  |
| Other pulmonary | 107 |  | |  |
| Ophthalmologic | 116, 117, 118, 119, 120, 121, 122, 123, 124 |  | |  |
| Ear, nose, and throat | 125, 126, 127 |  | |  |
| Other renal | 128, 129, 130, 132 |  | |  |
| Genitourinary | 133, 134, 136, 137, 138, 139, 140 |  | |  |
| Obstetric | 141, 142, 143, 144, 145, 146, 147 |  | |  |
| Other dermatologic | 150, 151, 153 |  | |  |
| Injury excluding hip fracture | 154, 155, 156, 157, 159, 160, 161, 162 (Excluded ICD-9-CM codes on right) | Exclude 162.9, 162.3, 162.5, 162.8, 162.2, 162.4 | |  |
| Symptoms (major and minor) excluding respiratory | 166, 167 (Excluded ICD-9-CM codes on right) | Exclude 780.2, 786.0, 786.00, 786.01, 786.02, 786.05, 786.06, 786.50, 786.59, 786.9, 780.2 | |  |
| Neonatal | 168, 169, 170, 171, 172 |  | |  |
| Organ transplant | 173, 174, 175 |  | |  |
| Miscellaneous surgical and non-surgical procedures | 176, 177, 178, 179, 180, 181, 182 |  | |  |
| Durable medical equipment | 185, 186, 187, 188, 189 |  | |  |
| Other | 183, 184 |  | |  |
| Skin ulcer including decubitus ulcer | 148, 149 |  | |  |
| Complications of care | 163, 164, 165 |  | |  |
| CMS: Centers for Medicare & Medicaid Services; ICD-9-CM: International Classification of Diseases, Ninth Revision, Clinical Modification | | | |  |
