# Supplementary material for: Readmissions after Hospitalization for Heart Failure, Acute Myocardial Infarction, or Pneumonia among Young and Middle-Aged Adults: A Retrospective Observational Cohort Study
Source: PLoS Med. 2014 Sep 30;11(9):e1001737. doi: 10.1371/journal.pmed.1001737 (PMC4181962; doi:10.1371/journal.pmed.1001737)
Supplement: Table S3 — Modified Condition Category codes for cardiovascular diagnoses. (DOCX) [file pmed.1001737.s003.docx]

| Table S3: Modified Condition Category codes for cardiovascular diagnoses. |
| --- |
|  |
| Modified condition category codes for cardiovascular diagnoses |
| 1 - Heart failure |
| 2 - Acute myocardial infarction |
| 3 - Unstable angina and other acute ischemic heart disease |
| 4 - Chronic angina and coronary artery disease |
| 5 - Valvular/rheumatic heart disease |
| 6 - Other cardiac disease including congenital heart and hypertensive disease |
| 7 - Arrhythmias and conduction disorders |
| 9 - Chest pain |
| 10 - Syncope |
| 11 - Acute stroke/transient ischemic attack |
| 12 - Pulmonary embolism/deep venous thrombosis |
| 13 - Other peripheral vascular disease |
