# Supplementary material for: Readmissions after Hospitalization for Heart Failure, Acute Myocardial Infarction, or Pneumonia among Young and Middle-Aged Adults: A Retrospective Observational Cohort Study
Source: PLoS Med. 2014 Sep 30;11(9):e1001737. doi: 10.1371/journal.pmed.1001737 (PMC4181962; doi:10.1371/journal.pmed.1001737)
Supplement: Table S4 — Modified Condition Category codes for pulmonary diagnoses. (DOCX) [file pmed.1001737.s004.docx]

| Table S4: Modified Condition Category codes for pulmonary diagnoses. |
| --- |
|  |
| Modified condition category codes for pulmonary diagnoses |
| 8 - Pleural effusion/pneumothorax |
| 14 - Cardio-respiratory failure |
| 15 - Chronic obstructive pulmonary disease/asthma |
| 16 - Pneumonia including aspiration pneumonitis |
| 25 - Fibrosis of lung and other chronic lung disorders |
| 28 - Other lung disorders including acute, congenital, and unspecified lung abnormalities |
| 29 - Primary cancer of the trachea, bronchus, lung, and pleura |
