# Supplementary material for: Readmissions after Hospitalization for Heart Failure, Acute Myocardial Infarction, or Pneumonia among Young and Middle-Aged Adults: A Retrospective Observational Cohort Study
Source: PLoS Med. 2014 Sep 30;11(9):e1001737. doi: 10.1371/journal.pmed.1001737 (PMC4181962; doi:10.1371/journal.pmed.1001737)
Supplement: Table S5 — Adjusted 30-day risk of readmission with the addition of length of index hospital stay as a measure of disease severity. (DOCX) [file pmed.1001737.s005.docx]

**Table S5: Adjusted 30-day risk of readmission with the addition of length of index hospital stay as a measure of disease severity**

|  |  | **30-Day Risk of Readmission (HR, 95% CI)** | | | |
| --- | --- | --- | --- | --- | --- |
| **Cohort** | **Model** | **18-39 yrs** | **40-54 yrs** | **55-64 yrs** | **≥65 yrs (referent group)** |
| HF | Adjusted for race, payer, comorbidities | 1.12  (1.05-1.20) | 1.01  (0.97-1.05) | 0.98  (0.95-1.01) | 1.00 |
|  | + adjustment for Length of stay | 1.12  (1.05-1.19) | 1.00  (0.97-1.04) | 1.12  (1.05-1.20) | 1.00 |
| AMI | Adjusted for race, payer, comorbidities | 0.81  (0.70-0.94) | 0.92  (0.86-0.98) | 0.92  (0.88-0.97) | 1.00 |
|  | + adjustment for Length of stay | 0.82  (0.70-0.95) | 0.91  (0.86 -0.97) | 0.92  (0.87-0.97) | 1.00 |
| Pneumonia | Adjusted for race, payer, comorbidities | 0.87  (0.81-0.92) | 0.99  (0.95-1.04) | 0.99  (0.95-1.02) | 1.00 |
|  | + adjustment for Length of stay | 0.86  (0.81-0.92) | 0.98  (0.94 -1.02) | 0.98  (0.94-1.02) | 1.00 |
